# Psychological distress and loneliness among European university students during the COVID-19 pandemic: a European survey

**DOI:** 10.1186/s12889-025-25001-3

**Published:** 2025-11-07

**Authors:** Caixin Tian, Roberto Ariel Abeldaño Zuñiga, Moréniké Oluwátóyìn Foláyan, Jarno Tuominen, Jorma I. Virtanen

**Affiliations:** 1 Faculty of Medicine, University of Bergen, Bergen, Norway; 2 Faculty of Social Sciences, University of Helsinki, Helsinki, Finland; 3 Department of Child Dental Health, Obafemi Awolowo University, Ile-Ife, Nigeria; 4 Department of Psychology and Speech-Language Pathology, University of Turku, Turku, Finland; 5 Faculty of Medicine, University of Turku, Turku, Finland

**Keywords:** Psychological distress, Loneliness, Students, COVID-19, Europe

## Abstract

**Background:**

The COVID-19 pandemic and various consequent isolation, quarantine and curfew measures to curb the spread of the virus caused an increase in anxiety, depression, and loneliness among university students. The aim of this study was to assess the association between psychological distress and loneliness among university students in Europe during the COVID-19 pandemic.

**Methods:**

This cross-sectional study was part of the COVIDiSTRESS global survey conducted during the first wave of COVID-19 pandemic in 2020. We evaluated for psychological stress and loneliness among university students in Europe (*n* = 11 231) using the Perceived Stress Scale (PSS-10) and the Loneliness Scale (SLON-3). The associations between the students’ psychological stress levels, loneliness, and perceived helplessness were analysed using chi-square test, ANOVA, t-test, and regression models.

**Results:**

In total, 9737 university students (69.9% female) between 18 and 34 years (mean: 25.3 years from 13 European countries were included in the analyses. Most (89.6%) of the university students experienced moderate stress levels, with women reporting significantly more stress than men (*p* < 0.001). Differences in stress levels were also associated with geographic region, marital status, and age. Higher levels of psychological stress were associated with the students’ loneliness levels; loneliness accounted for 12.9% of the variance in stress. Perceived helplessness was associated with loneliness and gender and country of residence.

**Conclusions:**

Majority of the university students in Europe experienced increased level of stress and loneliness during the first wave of COVID-19. Perceived helplessness was significantly associated with stress and correlated with gender, various loneliness factors and country of origin.

## Background

COVID-19, caused by Severe Acute Respiratory Syndrome Coronavirus-2 (SARS-CoV-2), was declared a pandemic by the World Health Organization (WHO) on March 11, 2020 [1]. The pandemic significantly affected people’s physical and mental health [2–4]. Factors explaining these findings include, the unpreparedness of individuals and communities for the impact of the disease [5], and the toll the preventive measures taken to control the pandemic placed on the mental health [6]: the large-scale travel restrictions, mandatory quarantine policies, curfews, and lockdowns caused distress and depressive symptoms [7–9].

Young people were also affected by the psychosocial effects of the pandemic. Social and family isolation and changes to lifestyles and daily habits led to distress and loneliness [10–12]. The closure of campus services as a preventive measure for COVID-19 left many university students confined to their dormitories or homes, with less contact with their peers and fewer social activities. Students’ social lives moved online to maintain social bonds and address social needs; a recent meta-analysis found a small overall negative effect of social media use on mental health with high heterogeneity between studies [13]. These negative psychosocial effects of the pandemic were globally greater for younger people and students than in Europe [14–16]. However, little is known about the level of psychological stress and loneliness among university students’ in Europe during the pandemic and related associations.

During the pandemic, understanding the determinants of individual behaviour became an essential question, as the spread of the disease and the actions to mitigate it (e.g. vaccinations) were contingent on individual and collective choices. Previous research shows that changing behaviours is notoriously difficult, even when the behaviour in question is relatively simple (for example handwashing distancing) [16] or when we hold every intention to change our behaviour (i.e. the intention-action gap or the value-action gap) [17]. The Self-Determination theory of motivation proposes that people strive to balance three psychological basic needs: need of autonomy, need for competence, and need to belong [18]. The theory suggests that people’s behaviour is shaped by their intentions, which are influenced by attitudes and beliefs. The pandemic likely restricted students’ opportunities for social interaction (e.g. reduced physical access to friends), potentially leading to heightened stress and loneliness as they lacked social opportunities and motivation to engage in typical coping behaviours.

Furthermore, the pandemic increased feelings of uncertainty (i.e. decreased sense of control and autonomy) which can either lead to increased passivity (for example, via negative emotions or stress), or alternatively to attempts to regain control by acting in an agentic manner (i.e. deciding to take up the vaccine, but also via reactance, i.e. acting in a manner to regain autonomy by for example defying public policies) [19–23]. Furthermore, most of our behaviours are socially determined, by for example, what I believe my neighbour to do or by considerations of what impact a specific behaviour has for social status. During the pandemic such social benchmarking was lost and due to restrictions behaviour did not necessarily impact social standing.

In conclusion, the key psychological needs of students were under strain during the pandemic, which likely leads to both more problematic experiential and behavioural outcomes. Younger people and students have generally experienced higher negative emotions during the pandemic than older individuals and this has been specifically pronounced in Europe [14]. However, little is known about European university students’ psychological stress and loneliness during the pandemic. Thus, our study aims to assess the stress and loneliness levels among the European university students during the COVID-19 pandemic.

## Materials and methods

This was a cross-sectional study based on the COVIDiSTRESS Global Survey was conducted between 30 March and 30 May 2020 during the first wave of the COVID-19 pandemic (https://osf.io/2ftma/). The COVIDiSTRESS global survey (COVIDiSTRESS global survey network, 2020) is a large-scale study of the psychological impact of COVID-19 conducted in 179 countries in six continents. The COVIDiSTRESS global survey collected data through online platforms (university networks, social media, and institutional mailing lists) (2020). There were 173,426 participants recruited by over 150 researchers using 48 languages and dialects [23]. The participants were at least 18 years of age, and all signed written consent for study participation. The survey was designed to capture diverse populations across 179 countries. For this study, we specifically extracted data from European university students (aged 18–34 years) to address our research objectives. Ethical approval for the study was obtained from Aarhus University, Denmark, with formal approval granted in June 2020 (# 2019-616-000009).

This study extracted the data of respondents from 13 countries (Bulgaria, Croatia, the Czech Republic, Denmark, Finland, France, Germany, Italy, Kosovo, Lithuania, Poland, Portugal, and Turkey) in three European regions (Northern Europe, Central Europe and Southern Europe). For this study we defined a minimum participation rate per country (≥ 200 participants) and multilingual accessibility to ensure broad representation (Table 1). Data was collected using questionnaires translated into 13 languages native to the countries in which the respondents lived (Albanian, Bulgarian, Croatian, Czech, Danish, Finnish, French, German, Italian, Lithuanian, Polish, Portuguese, and Turkish). Subtle language differences were adapted to the national context, and novel translations underwent a backtranslation- procedure [23]. For this analysis, the 13 countries were classified into the three geographical regions: Northern Europe (Denmark and Finland, *N* = 3105), Central Europe (France, Germany, Lithuania, Poland, the Czech Republic, and Kosovo, *N* = 4701) and Southern Europe (Portugal, Italy, Croatia, Bulgaria, and Turkey, *N* = 1931).

### Dependent variable

The dependent variable was distress, measured using the 10-item Perceived Stress Scale (PSS-10) developed by Cohen et al. (1983) [24]. Respondents rated how often they experienced certain feelings and thoughts over the past week on a five-point Likert scale (0 = never to 4 = very often). The scale includes 10 questions, such as feeling upset by unexpected events or unable to control important things. Four questions (4, 5, 7, 8) are positively stated and were reverse-scored. The total PSS-10 score ranges from 0 to 40, with higher scores indicating greater perceived stress. Scores of 0–13 indicate low stress, 14–26 moderate stress, and 27–40 high stress.

The PSS-10 has two subscales. The perceived helplessness subscale of the PSS-10, strongly linked to mental health issues [25], includes six questions (pss1, pss2, pss3, pss6, pss9 and pss10). Respondents were asked how often they experienced the following feelings over the past week: (1) upset by unexpected events, (2) unable to control important things, (3) nervous and stressed, (4) unable to cope with tasks, (5) angered by uncontrollable events, and (6) overwhelmed by difficulties. This sub-scale had a Cronbach’s alpha score of 0.836 for the current study.

The second scale is the “lack of self-efficacy subscale” that has four items (pss4, pss5, pss7 and pss8). This scale had a Cronbach’s alpha score of 0.726 in the current study.

### Independent variables

Loneliness and concern over the coronavirus were independent variables. Loneliness was measured by the 3-item UCLA Short Loneliness Scale (SLON-3) developed by Hughes et al. [26], which assesses feelings of lacking companionship, feeling left out, and feeling isolated. Each SLON-3 item was rated on a 3-point scale (1 = Hardly ever; 3 = Often), with scores ranging from 3 to 12. The total score (SLON-3 total) for each participant is the sum of the ratings for the three items, yielding a possible score range of 3 to 12. Higher scores indicate greater levels of loneliness.

Concern over the coronavirus was measured using a 5-item scale from the COVIDiSTRESS survey [24], where respondents rated their concerns on a 6-point Likert scale (1 = Strongly Disagree; 6 = Strongly Agree) about the impact of COVID-19 on themselves, their family, friends, country, and globally. The total score for the COVID concerns is the sum of the ratings for the five items. The questions included in this scale were: How much do you agree that you are you concerned about the consequences of the Coronavirus for yourself? for your family? for your close friends? for your country? For other countries across the globe?

### Confounding variables

Respondents’ age, gender, nationality, country of residence, educational background, and marital status were included as confounding variables. The data collected from the respondents’ gender was categorised as male, female or other/would rather not say. The respondents’ educational status was categorised as (1) PhD/Doctorate, (2) College degree, (3) Some College or equivalent, (4) Up to 12 years of school, (5) Up to 9 years of school, (6) Up to 6 years of school, (7) None. The categories were later combined to Higher education (PhD/Doctorate/College degree), Medium education (Some college or equivalent), Low education (School 12 years or less).

### Statistical analysis

The variables were analysed using descriptive statistics, mean and standard deviations for quantitative variables, and frequencies and percentages for categorical variables. The bivariate analyses included Chi^2^-test for comparing prevalences, and t-tests or Anova for comparing means between two or three groups, respectively.

Finally, after verifying normal distribution, three linear regression models were employed as follows. The assumptions of linear regression were tested and confirmed to be met.

- Model 1: The dependent variable was “perceived helplessness.”
- Model 2: The dependent variable was “lack of self-efficacy.”
- Model 3: The dependent variable was the total score on the perceived stress scale (PSS).

The explanatory variables for all three models included age, residing outside the home country, gender, SLON 1, SLON 2, SLON 3, and the total score on the COVID concern scale. Each model focuses on distinct dimensions of stress to capture the complexity of stress perception. Model 1 investigates “perceived helplessness” to specifically measure the lack of control aspect of stress. Model 2 focuses on “lack of self-efficacy,” which complements helplessness by targeting a different stress facet, where individuals feel ineffective. Model 3 aggregates these into the total PSS score, providing an overall measure of perceived stress, which offers insight into the combined impact of stress dimensions. While these subscales are correlated (as expected for components of stress), they represent theoretically distinct dimensions of stress perception [24]. Separate analyses allow us to identify unique predictors for each dimension, as well as to avoid masking divergent relationships that could inform targeted intervention.

On the other hand, the explanatory variables were selected based on their theoretical relevance to stress response and demographic factors. Age, residing outside the home country, and gender are essential demographic factors known to influence stress experiences. Loneliness (SLON 1, 2, and 3) likely represents specific stressors or categories of concern that are particularly relevant to the study population. Lastly, COVID concern scale total score was included to capture pandemic-related concerns, a potentially impactful factor during this period. The statistical analyses were performed using IBM SPSS Statistics for Windows version 28.0 software.

## Results

Table 1 shows the main characteristics of the European university students by the stress components. The final sample consisted of 9,737 individuals. Of these, 70.0% were female (*n* = 6,806); 31.9% were 18 to 24 years, and 48.3% were 25 to 29 years old. Regarding their countries of residence during the pandemic, 41.0% were in the Northern European countries, and 41.7% were in the Central European countries. Additionally, 18.3% of the participants were residing outside their home country. In terms of educational background, 81.7% reported some medium education, while 18.3% had higher education.

**Table 1:** Main characteristics of the participants by the stress components (*n* = 9737)

Variables	Categories	*n*	%	Perceived helplessness			Lack of self-efficacy			Total stress score**		
				Mean	SD	*P* value***	Mean	SD	*P* value***	Mean	SD	*P* value***
Gender	Female	6806	69,9	12.01	5.00	< 0.001	8.07	2.08	< 0.001	20.09	3.09	< 0.001
	Male	2730	28,0	9.03	5.01		9.07	3.00		19.00	3.09	
	Other/would rather not say	201	2,1	12.08	5.05		8.00	2.09		20.09	4.01	
Age Groups	18 to 24	3105	31,9	11.03	5.02	0,465	8.09	2.09	< 0.001	20.02	4.00	< 0.001
	25 to 29	4701	48,3	11.04	5.02		9.02	2.09		20.07	3.09	
	30 to 34	1931	19,8	11.03	4.09		9.03	2.08		20.07	3.06	
European region*	Northern Europe	3996	41,0	11.02	5.02	< 0.001	9.02	2.08	< 0.001	20.04	3.08	< 0.001
	Central Europe	4056	41,7	11.00	5.03		9.01	2.09		20.01	4.02	
	Southern Europe + Turkey	1575	16,2	12.04	4.09		8.04	2.09		20.09	3.07	
Education Background	Higher education	1779	18,3	11.02	5.03	0.005	9.01	2.09	< 0.001	20.04	3.09	0,072
	Medium education	7958	81,7	11.03	5.02		8.09	2.08		20.03	4.00	
	Low education	1575	16,2	11.07	5.02		8.07	2.09		20.05	4.00	
Are you residing outside home country?	Yes	1779	18,3	11.05	5.01	0,102	9.02	2.09	< 0.001	20.03	4.00	< 0.001
	No	7958	81,7	11.03	5.02		8.09	2.09		20.08	3.09	

*Countries with 250 respondents or more included

** PSS-10 = Perceived Stress Scale. *N* = number of participants who completed the scale. % = proportion of participants who completed the scale

*** Chi-square tests were employed

Females had a higher mean score in perceived helplessness (12.01, SD = 5.0) when compared to the males (9.03, SD = 5.01) (*p* < 0.001). Males showed a higher mean score in lack of self-efficacy than females (9.07, SD = 3.00 and 8.07, SD = 2.08, respectively) (*p* < 0.001). Females had a higher total score on the PSS scale than males (mean = 20.09, SD = 3.09 and mean = 19.00, SD = 3.09, respectively) (*p* < 0.001).

Table 2 illustrates the European university students’ loneliness scores and its three components, and COVID concerns by perceived stress group. The mean SLON-3 values of the low perceived stress, moderate perceived stress and high perceived stress groups were 4.31, 5.61 and 7.09, respectively. The SLON-3 mean values increased consistently and significantly from low perceived stress group to moderate and high perceived stress groups (*p* < 0.001). When the university students’ COVID concerns were analysed using the total scores of the COVID concern scale by perceived stress group, similar significant findings were seen (*p* < 0.001).

**Table 2:** The European university students’ loneliness SLON-3 scores and its components (mean, SD) by perceived stress (PSS-10) and COVID concern*

Explanatory variables	Outcome groups	*N*	Mean	Std. Deviation	*p* value**
SLON 1	Low perceived stress	388	1.562	0.767	< 0.001
	Moderate perceived stress	8727	1.945	0.840	
	High perceived stress	622	2.384	0.792	
	Total	9737	1.958	0.845	
SLON 2	Low perceived stress	388	1.186	0.468	< 0.001
	Moderate perceived stress	8727	1.570	0.756	
	High perceived stress	622	2.140	0.860	
	Total	9737	1.591	0.770	
SLON 3	Low perceived stress	388	1.567	0.766	< 0.001
	Moderate perceived stress	8727	2.097	0.837	
	High perceived stress	622	2.566	0.684	
	Total	9737	2.106	0.840	
SLON-3 total	Low perceived stress	388	4.314	1.583	< 0.001
	Moderate perceived stress	8727	5.612	1.938	
	High perceived stress	622	7.090	1.815	
	Total	9737	5.655	1.970	
COVID concern: total score*	Low perceived stress	388	20.255	5.261	< 0.001
	Moderate perceived stress	8727	22.188	4.530	
	High perceived stress	622	24.095	4.345	
	Total	9737	22.233	4.591	

Compilation based on COVIDiSTRESS global survey data

*Abbreviations*: *SLON-3* Loneliness Scale, N number of participants who completed the scale, *Mean* scale mean. *Std. Deviation* standard deviation

*COVID concern = total score

*SLON 1 = “In the last week, how often have you been felt that you lacked companionship”. SLON 2 = “In the last week, how often have you been felt left out”. SLON 3 = “In the last week, how often have you been felt isolated from others”

**ANOVA tests were employed

The multiple linear regression model examining perceived helplessness explained 32.9% of the variance (Adjusted R² = 0.329). Age was negatively associated with perceived helplessness (B = −0.002, 95% CI: −0.017, 0.013, *p* = 0.779), although this effect was small and non-significant. Table 3 shows that individuals residing outside their home country reported higher levels of helplessness (B = 0.379, 95% CI: 0.175, 0.583, *p* < 0.001), as did females (B = 1.099, 95% CI: 1.503, 1.859, *p* < 0.001) and those identifying with other genders (B = 2.291, 95% CI: 1.745, 2.836, *p* < 0.001), compared to males.

**Table 3:** Linear regression coefficients for estimating perceived helplessness, lack of self-efficacy, and the total score of the PSS-10 scale

Variables	Perceived helplessness							Lack of self-efficacy							Total PSS-10						
	Std Coefficients B	Unstd Coefficients B	95,0% Confidence Interval for B		P-value**	Collinearity stats		Std Coefficients B	Unstd Coefficients B	95,0% Confidence Interval for B		P-value**	Collinearity stats		Std Coefficients B	Unstd Coefficients B	95,0% Confidence Interval for B		P-value**	Collinearity stats	
			Lower Bound	Upper Bound		Tolerance	VIF			Lower Bound	Upper Bound		Tolerance	VIF			Lower Bound	Upper Bound		Tolerance	VIF
Age	−0.019	−0.010	−0.017	−0.002	0.013	0.973	1.028	0.126	0.036	0.032	0.041	< 0.001	0.973	1.028	0.068	0.027	0.020	0.033	< 0.001	0.973	1.028
Are you residing outside home country? (yes vs. no)	0.027	0.379	0.175	0.583	< 0.001	0.978	1.022	0.015	0.119	−0.007	0.245	0.063	0.978	1.022	0.047	0.498	0.324	0.672	< 0.001	0.978	1.022
Gender (females vs. males)	0.146	1.681	1.503	1.859	< 0.001	0.895	1.117	−0.071	−0.465	−0.575	−0.356	< 0.001	0.895	1.117	0.136	1.215	1.064	1.367	< 0.001	0.895	1.117
Gender (others vs. males)	0.063	2.291	1.745	2.836	< 0.001	0.938	1.066	−0.054	−1.105	−1.440	−0.769	< 0.001	0.938	1.066	0.042	1.186	0.772	1.650	< 0.001	0.938	1.066
SLON 1*	0.119	0.732	0.624	0.841	< 0.001	0.680	1.470	−0.081	−0.284	−0.351	−0.217	< 0.001	0.680	1.470	0.094	0.448	0.356	0.541	< 0.001	0.680	1.470
SLON 2*	0.306	2.068	1.953	2.184	< 0.001	0.736	1.359	−0.255	−0.979	−1.050	−0.908	< 0.001	0.736	1.359	0.208	1.089	0.991	1.187	< 0.001	0.736	1.359
SLON 3*	0.179	1.099	0.989	1.210	< 0.001	0.658	1.521	−0.156	−0.544	−0.612	−0.475	< 0.001	0.658	1.521	0.117	0.556	0.461	0.650	< 0.001	0.658	1.521
COVID concern (total score)	0.154	0.169	0.153	0.186	< 0.001	0.958	1.044	−0.084	−0.052	−0.062	−0.042	< 0.001	0.958	1.044	0.138	0.117	0.103	0.131	< 0.001	0.958	1.044
	Adj. R2: 32.9%							Adj. R2:21.2%							Adj. R2: 18.5%						

*SLON 1 = “In the last week, how often have you been felt that you lacked companionship”. SLON 2 = “In the last week, how often have you been felt left out”. SLON 3 = “In the last week, how often have you been felt isolated from others”. ** T-test** STD (Standardized); Unstd (Unstardardized). *VIF* Variance Inflation Factor

Higher scores on the SLON subscales were strongly associated with increased perceived helplessness, with SLON 1 (B = 0.732, 95% CI: 0.624, 0.841, *p* < 0.001), SLON 2 (B = 2.068, 95% CI: 1.953, 2.184, *p* < 0.001), and SLON 3 (B = 1.099, 95% CI: 0.989, 1.210, *p* < 0.001) all showing significant positive relationships. Similarly, concern about Covid-19 was a significant predictor, with higher levels of concern associated with increased perceived helplessness (B = 0.169, 95% CI: 0.153, 0.186, *p* < 0.001).

The regression model for lack of self-efficacy explained 21.2% of the variance (Adjusted R² = 0.212). Age was positively associated with lower self-efficacy (B = 0.036, 95% CI: 0.032, 0.041, *p* < 0.001). Residing outside one’s home country showed a marginal effect (B = 0.119, 95% CI: −0.007, 0.245, *p* = 0.063), which was not statistically significant. Females (B = −0.465, 95% CI: −0.575, −0.356, *p* < 0.001) and individuals identifying with other genders (B = −1.105, 95% CI: −1.440, −0.769, *p* < 0.001) reported significantly lower self-efficacy compared to males.

The SLON subscales were negatively associated with self-efficacy: SLON 1 (B = −0.284, 95% CI: −0.351, −0.217, *p* < 0.001), SLON 2 (B = −0.979, 95% CI: −1.050, −0.908, *p* < 0.001), and SLON 3 (B = −0.544, 95% CI: −0.612, −0.475, *p* < 0.001). Concern about Covid-19 was also negatively associated with self-efficacy (B = −0.052, 95% CI: −0.062, −0.042, *p* < 0.001).

The total PSS10 scale regression model explained 18.5% of the variance (Adjusted R² = 0.185). Age was positively associated with higher perceived stress (B = 0.027, 95% CI: 0.020, 0.033, *p* < 0.001). Living outside the home country was significantly associated with increased perceived stress (B = 0.498, 95% CI: 0.324, 0.672, *p* < 0.001). Females (B = 1.215, 95% CI: 1.064, 1.367, *p* < 0.001) and individuals of other genders (B = 1.186, 95% CI: 0.772, 1.650, *p* < 0.001) also reported significantly higher stress compared to males.

Higher scores on the SLON subscales were associated with higher perceived stress, with SLON 1 (B = 0.448, 95% CI: 0.356, 0.541, *p* < 0.001), SLON 2 (B = 1.089, 95% CI: 0.991, 1.187, *p* < 0.001), and SLON 3 (B = 0.556, 95% CI: 0.461, 0.650, *p* < 0.001) all showing significant positive relationships. Concern about Covid-19 also significantly predicted higher perceived stress (B = 0.117, 95% CI: 0.103, 0.131, *p* < 0.001).

## Discussion

Our study found that the majority of the European university students had moderate stress and loneliness during the first wave of the COVID-19 pandemic. Women reported significantly higher levels of stress in comparison to men. European university students residing outside their country of origin reported higher levels of stress compared to those residing in their home country. The students who reported their marital status as other or would not say perceived more often high stress. Younger university students reported more often low levels of stress. Perceived helplessness was associated with loneliness and gender, and country of residence. Central Europe had the highest percentage of low-stress participants, whereas Northern Europe, Southern Europe and Turkey had a higher number of moderate- and high-pressure participants. University students with higher perceived stress levels reported significantly higher loneliness and COVID-19 concerns. As perceived stress increased, the level of loneliness increased too: those in the high perceived stress group experiencing the greatest loneliness. These findings highlight that gender, SLON factors and residency status have strong, significant impacts on perceived helplessness.

During the first phase of the COVID-19 pandemic, over 80% of the European university students experienced distress from infection risks, while over 70% were concerned about the well-being of distant friends or relatives. More than 60% were distressed over work performance and social activity limitations, and over 50% felt lonely. Younger students reported more low stress which contradicts previous studies in Europe [14]. Our study revealed significant levels of psychological distress in various regions. A significant portion of participants reported experiencing increased levels of stress and anxiety caused by various factors, such as the fear of catching an infection, worries about the well-being of relatives, economic uncertainties, and disruptions to daily routines. The considerable levels of psychological distress observed underscore the necessity for integrating mental health support into public health responses to pandemics. The analysis revealed a weak correlation (R² = 0.129) between loneliness and stress among the university students indicating that loneliness accounted for approximately 12.9% of the variance in stress levels. However, the observed correlation between loneliness and stress suggests the presence of potential moderating or mediating factors. For instance, gender differences may play a moderating role, as previous literature indicates that women may experience and cope with loneliness differently than men [27]. Lack of local social especially support among students living abroad and COVID-19 related concerns may also mediate perceived stress. While these models identified statistically significant predictors of stress dimensions, some effects were modest in magnitude (COVID-19 concerns: B = 0.117 for total stress). Small effects may still be practically meaningful at a population level, particularly for high-prevalence issues like loneliness. For instance, the strong association between SLON-2 and helplessness (B = 2.068) suggests that targeted social inclusion programs could disproportionately benefit students’ emotional well-being. Conversely, the limited variance explained in total stress (Adj. R² = 18.5%) underscores the multifactorial nature of pandemic-related distress, warranting holistic interventions beyond psychosocial support.

Research has shown that COVID-19 has influenced mental health, but mental health status has also affected the compliance with COVID-19 preventive measures [27–29]. People with good mental health were more likely to comply with the pandemic prevention measures while people who felt lonely were less likely to comply with pandemic prevention measures [30].

Perceived helplessness measures an individual’s feelings of a lack of control over the circumstances or own emotions or reactions [25]. The various factors e.g. age, gender, and residence country exhibit heterogeneous associations with perceiving helplessness compared to perceived stress. In addition, those who were single, divorced or widowed reported higher levels of perceived helplessness in line with earlier studies [31]. This suggests that helplessness is particularly sensitive to these factors. Conversely, the use of perceived distress as a broad measure may obscure specific associations that only become apparent when individual dimensions are examined.

Lack of efficacy refers to an individual’s diminished confidence in their ability to cope with challenges and achieve desired outcomes. Our study showed that female students experienced higher self-efficacy than male students, a finding which is contrary to previous study [32]. In addition, the older students reported higher lack of self-efficacy as found earlier [33]. The implications of these findings emphasise the need for targeted interventions to improve self-efficacy, particularly among vulnerable groups. Addressing the specific needs of those with lower self-efficacy can improve mental health outcomes.

Our study found that increased loneliness was associated with increased levels of distress, a finding that is consistent with previous research [34, 35]. Loneliness not only directly affects an individual’s mental health but may also further affect psychological states through increased feelings of stress. The effects of loneliness may have been more pronounced during the COVID-19 pandemic. Social isolation and reduced face-to-face interactions during COVID-19 pandemic exacerbated individuals’ feelings of loneliness, which increased their distress levels [36].

Our study also showed that European university students’ loneliness increased significantly during the first phase of the pandemic and was strongly associated with high perceived stress levels (Table 2). Individuals with high levels of loneliness often lack effective social support networks, which may lead them to feel more helpless in the face of stressors [37].

The study showed that gender and place of residence significantly influenced loneliness, which is consistent with previous findings [38, 39], who found that gender and socio-environmental factors play an important role in the development of loneliness. Typically, women report higher levels of loneliness, which may be related to their social roles and expectations [40]. In addition, university students living abroad reported higher levels of loneliness, which may be related to being away from home, acculturation problems, and lack of local social support. Geographically, Central Europe had the highest percentage of low-perceived-stress participants, whereas Northern Europe, Southern Europe and Turkey had a greater amount of moderate- and high-pressure participants.

Considering our results from a theoretical perspective, our findings highlight how the basic needs, as described by the Self-Determination Theory [18] are dynamically interrelated to either increase or regulate distress. Perceived helplessness can be characterized by a disruption in both needs for competence and for autonomy, i.e., we do not either have the capacity or control to alter the circumstances. Further, loneliness (i.e. perceived social isolation) can be seen as an experiential indicator of our need to belong with others is not met. Our findings highlight how changes in these needs affects our perceived stress. There is a risk of a vicious cycle, as both social and environmental support is required to regulate these psychological basic needs and maintain well-being. In situations such as the COVID-19 pandemic which for many people impacted each of these needs at once while also disrupting existing support and coping strategies, the increase in psychological distress is understandable.

In summary, our findings contribute to the evidence of the psychological impact of the COVID-19 pandemic on university students in Europe. We observed that female university students and those living abroad reported significantly higher levels of stress and isolation, reinforcing previous research [27, 41] that has emphasized the impact of gender and social context on mental health during crises.

### Limitations

A limitation of this study is its reliance on self-report data, which has the potential for response bias. Self-report data rely on participants’ subjective perceptions and interpretations of their experiences, behaviours and attitudes, which may be influenced by various factors such as social desirability bias, recall bias and response style. Participants may provide responses that they perceive to be socially acceptable or desirable, resulting in inflated or distorted estimates of the variables under investigation. Similarly, recall bias may affect the accuracy of participants’ recollections of past events or experiences, resulting in inaccuracies in reported data.

Furthermore, the focus of this study on college students represents another limitation, as it may limit the generalizability of the findings to other populations. College students constitute a specific demographic group with unique characteristics, experiences, and perspectives that may differ from the general population. Consequently, conclusions drawn from this sample may not represent broader populations, such as non-student adults or adolescents. This limitation impairs the external validity or generalizability of the findings, as they may not apply or transferable to other populations or settings.

To mitigate these limitations, future research efforts could employ a variety of methods, including longitudinal designs, experimental studies, and mixed methods, to supplement self-reported data and enhance the robustness and validity of findings. Furthermore, expanding the study sample to a more diverse and representative population beyond college students will allow researchers to draw more general conclusions about the psychological impact of the COVID-19 pandemic across different demographic groups and contexts. This will increase the applicability and relevance of the findings to inform targeted interventions and support mechanisms to address the psychological consequences of the global health crisis on a larger scale. 

## Conclusions

The study discovered elevated moderate stress and loneliness levels among the European university students during the first wave of the COVID-19 pandemic. Perceived helplessness was the significant factor for stress, and it was associated with gender, the SLON factors and residency status of the students.

## Data Availability

Data is provided within the manuscript (https://osf.io/2ftma/).

## Abbreviations

CI: Confidence interval
COVID-19: Coronavirus disease
SD: Standard deviation
SLON-3: UCLA Short Loneliness Scale
PSS-10: Perceived Stress Scale
WHO: World Health Organization
